# A 13-Year-Old with Coexistence of Gastric Volvulus and Leprosy: A Case Report of Two Rare Entities

**DOI:** 10.1155/2018/6125215

**Published:** 2018-08-14

**Authors:** Joanna Schneider, Rhett Mays

**Affiliations:** ^1^University of North Carolina School of Medicine, Chapel Hill, NC, USA; ^2^Mission Hospital, Asheville, NC, USA

## Abstract

Leprosy, or Hansen's disease, is caused by infection with *Mycobacterium leprae*. It is a rare diagnosis within the continental United States. We present the case of a 13-year-old immigrant from the Marshall Islands who presented with recurrent nausea, vomiting, and abdominal pain which are found to be due to intermittent gastric volvulus. Gastric volvulus is also exceedingly rare, with less than 8 pediatric cases on average per year. During her second hospitalization for recurrent acute gastrointestinal issues, nonspecific skin lesions were biopsied, revealing infection with *M. leprae*. The patient did not exhibit classic symptoms of leprosy but did have prominent skin changes including diffuse nodules. This case explores the pathophysiology connecting leprosy to volvulus, discussing the possible role of an inflammatory response to infection in causing gastric volvulus. The finding of lepromatous leprosy may have been unrelated but was fortuitous, as early intervention will result in avoidance of debilitating peripheral neuropathy and eventual disfiguration from Hansen's disease. This case highlights the importance of considering rare causes of gastric outlet obstruction including gastric volvulus and of considering leprosy in the differential for patients with unusual skin lesions or paresthesias who have a history of living or traveling in endemic areas.

## 1. Introduction

Leprosy, also known as Hansen's disease, is a chronic infectious disease caused by the acid-fast bacillus (AFB) *Mycobacterium leprae* [1]. It is rare in the United States (US), with less than 200 new cases per year [2]. Over half of patients diagnosed in the US were born elsewhere, with the Oceania region, which comprises Micronesia, contributing the largest number of total US cases [3]. In 2014, 14 of 175 total US cases (8.0%) were found in individuals born in Micronesia. Only 8 of the 175 cases (4.6%) were diagnosed in people under 16 years of age [4].

Leprosy typically affects the skin, peripheral nerves, and mucosal surfaces of the upper respiratory tract and eyes [5]. On an extensive literature review, we found no reported cases of leprosy presenting with gastric distension, splenomegaly, or gastric volvulus [1, 6–17].

Gastric volvulus is also an exceedingly rare diagnosis amongst children in the US. It occurs when the stomach twists about an axis and causes an obstruction which may be acute, recurrent, intermittent, or chronic. Even amongst causes of foregut obstruction, gastric volvulus is uncommon and is most commonly seen in adults with a paraesophageal hernia of the stomach. Between 1929 and 2007, 581 cases of gastric volvulus in children were found published in the English literature, yielding an average of less than eight cases per year [17].

This case report will discuss these two processes seen in a 13-year-old girl and whether or not there is likely a relationship between the two.

## 2. Case Presentation

A 13-year-old female of the Marshallese origin presented to the emergency department with complaints of nausea; nonbloody, nonbilious vomiting; and abdominal pain [18]. The patient denied any recent fevers, and no rash was reported. Review of systems was notable for the left ear drainage. Immunization status was unknown, and she was not reported to have been previously treated for any significant illness, though the history was limited by the patient's custodial circumstances. She lived in a small house in rural North Carolina with 21 other Marshallese immigrants and was cared for by relatives who had assumed care for her at the time of her immigration five years before.

**Figure 1:**
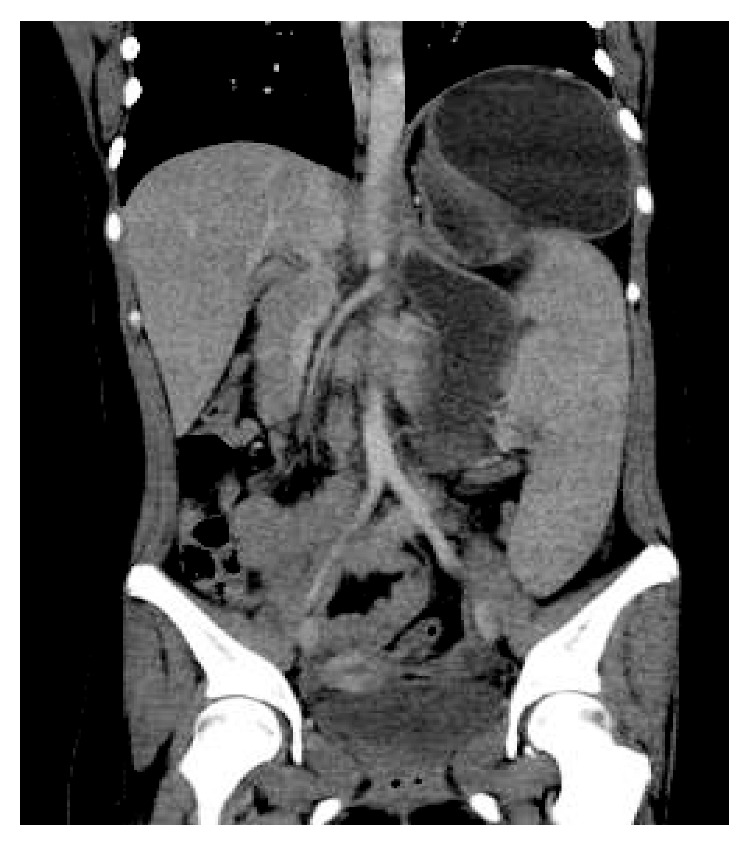
Coronal computed tomography scan of the abdomen showing marked gastric distension and splenomegaly.

Examination during the initial hospital visit revealed dehydration and acute otitis media with rupture of the tympanic membrane. Laboratory results revealed leukocytosis, prerenal azotemia, elevated liver enzymes, and mildly elevated lipase (Table 1). Computed tomography (CT) showed scattered focal pulmonary infiltrates, splenomegaly, and a markedly distended stomach without an obvious focus of mechanical obstruction (Figure 1). Cytomegalovirus and Epstein–Barr virus serology were requested with results suggesting prior exposure. She was admitted with a presumptive diagnosis of gastroparesis secondary to a nonspecific viral infection and possible mild pancreatitis. An NG tube was placed resulting in high volume output. Her symptoms gradually improved over several days with IV fluid support and bowel rest; her NG was successfully removed, and she was discharged home tolerating a regular diet.

**Figure 2:**
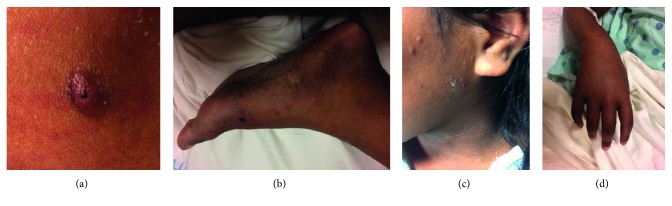
(a) Papular lesion with excoriations from itching on the patient's forearm. (b) The left foot with dark macules and papules. (c) Acanthosis nigricans around the patient's neck with dry, scaly papules along the lateral face. (d) The edematous hand with pigmented macules.

Two days after discharge, the patient returned with recurrence of her prior symptoms. The patient appeared acutely ill with dehydration. On lung exam, scattered crackles were noted. She was also noted to have diffuse small nodular lesions most apparent on her hands, feet, lower legs, and face (Figure 2). An advocate placed with the family reported her concern for additional symptoms of chronic weight loss and productive cough with posttussive emesis and reported the skin changes to have been present for months. Prior exposure to or testing for tuberculosis was unknown. The patient did not report symptoms of peripheral neuropathy.

**Figure 3:**
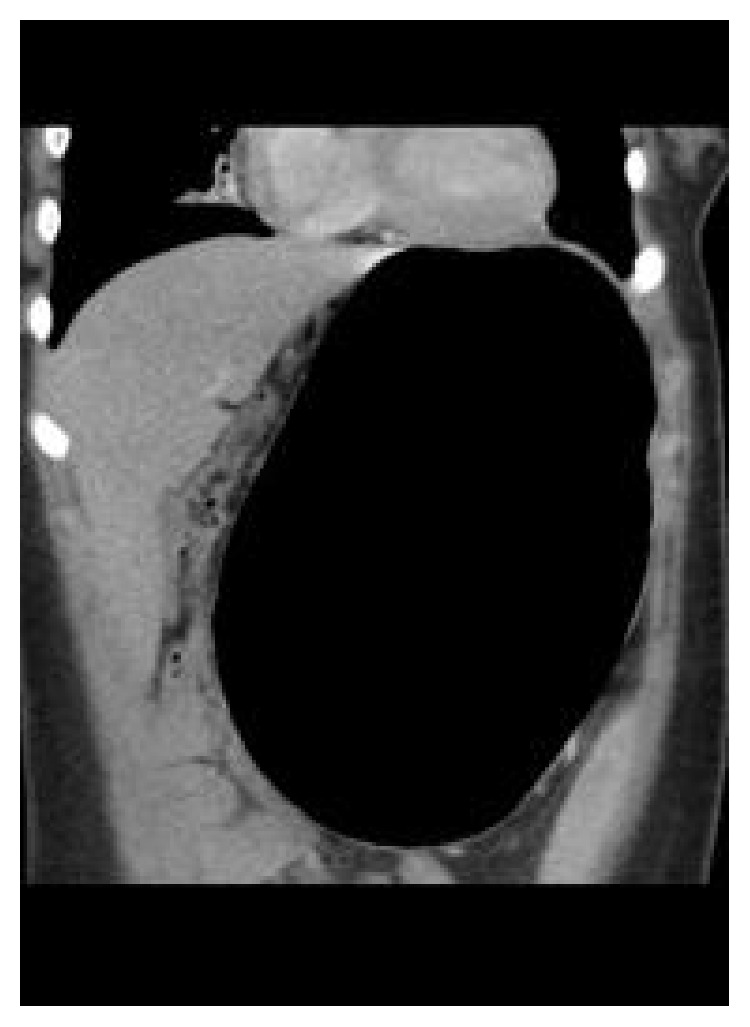
The coronal computed tomography scan of the abdomen showing persistent gastric dilatation.

Laboratory results revealed a relatively increased white blood cell count (21.4 × 10^9^ per liter) with significant worsening of her renal function (creatinine of 2.45 mg/dL which had previously been normalized with rehydration) and persistent mild elevation in liver enzymes and lipase. Respiratory viral screening was positive for rhino/enterovirus. Repeat CT imaging showed persistent gastric dilation and splenomegaly (Figure 3).

**Figure 4:**
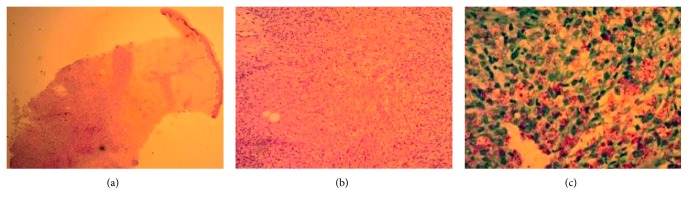
(a) Low-power image of epidermis, dermis, and subcutaneous tissue within the subcutaneous nodule showing granulomatous inflammation in the deep dermis and subcutis. (b) High-power image of the inflammation. (c) Fite's stain demonstrating the *M. leprae bacilli*.

The patient was placed on empiric antibiotics for community-acquired pneumonia and put on reverse isolation with concern for tuberculosis. Skin biopsies of multiple lesions were obtained and sent for pathology and culture (Figure 4). Because multiple attempts to place an NG tube failed and vomiting failed to respond to conservative measures, endogastroduodenoscopy was pursued. The scope passed into the stomach and through the pylorus easily, reaching a normal-appearing proximal duodenum. However, a feeding tube could not be passed beyond the pylorus and was left just proximal to the pylorus for decompression. Stomach mucosa was described as having a “cobblestone” appearance in places, with one area of ulceration possibly due to prior nasogastric tube. Stomach biopsies revealed *Helicobacter pylori* and chronic active gastritis, but were negative for AFB.

Due to persistent evidence of gastric outlet obstruction, an upper gastrointestinal study was performed which suggested gastric volvulus. The patient proceeded to gastropexy via open gastrostomy tube placement. The surgeons reported an extremely large and patulous stomach that had twisted mesenteroaxially. Gastric aspirates were negative for AFB.

Biopsies of the skin lesions confirmed the presence of many AFB with changes consistent with lepromatous leprosy due to *M. leprae* (Figure 4). Additionally, the serial sputum samples were positive for AFB, and culture of the skin sample for AFB showed evidence of growth within a few days, suggesting *Mycobacterium tuberculosis* (MTB) pulmonary infection and possibly disseminated MTB given selectivity of the culture medium.

In coordination with the health department and the infectious disease service, the patient was initiated on treatment for presumed disseminated MTB, noting that this would also provide coverage for *M. leprae*. However, polymerase chain reaction (PCR) testing from the sputum samples and skin samples failed to demonstrate MTB, while PCR testing performed at the Hansen's Disease Center of skin samples was positive for *M. leprae*. Retrospectively, it was felt that growth detected on the AFB culture was due to metabolism by an unusually large inoculum.

Gradually, the patient's oral intake improved. With hydration, her renal function again returned to normal. After several weeks of inpatient treatment, the patient discharged to complete therapy through the health department. Symptoms of gastric outlet obstruction have not returned.

## 3. Discussion

On initial presentation, the patient was treated presumptively for a self-limiting condition—viral or postviral gastroparesis—and seemed to respond appropriately. Though there was evidence of a systemic inflammatory process, it was not evident that she had any symptoms other than that of acute illness. It was not until her return and concurrent report of weight loss, chronic cough, and visualization of her subtle nodular skin disease that a more significant process was entertained. There was a high index of suspicion for disseminated MTB, with or without concurrent leprosy, prompted by likely exposure in this distinct patient population [19].

Despite skin pathology findings being classic for lepromatous leprosy, the presence of obstructive gastrointestinal symptoms, pulmonary infiltrates, and solid organ involvement suggested MTB. Endogastroduodenoscopy was performed anticipating evidence of duodenal obstruction from a tuberculous mass or stricture, as has previously been reported in the literature [20]. Even when no obstruction was visualized, falsely positive AFB cultures of the skin sample seemed to confirm the diagnosis, as *M. leprae* does not grow on AFB culture [21]. Indeed, it was not until true growth did not occur and both skin and respiratory samples were negative for MTB by PCR that empiric MTB treatment was stopped.

Though cases of leprosy that are acquired domestically remain rare, the Marshall Islands have the highest reported prevalence of leprosy worldwide [22]. Marshallese have the rights of the US citizens and have resettled widely in the US. While it is prudent to have a higher index of suspicion for this illness in Marshallese immigrants, it is also important to bear in mind that even amongst this population, leprosy remains uncommon with a prevalence of 55 per 10,000 [19].

The improbable concurrent diagnoses of gastric volvulus and leprosy initially led us to speculate that a prolonged gastroparesis in the setting of infection and systemic inflammation led to massive gastric distention and only secondarily to subsequent intermittent volvulus. However, there is no previously described association between leprosy and gastroparesis, and there is no direct evidence of gastroparesis in this patient. Surgical exploration revealed only massive gastric distention, which could have been either the cause or the effect of volvulus.

Leprosy is known to affect only peripheral nerve function, so we find it unlikely that active leprosy led directly to gastroparesis. Yet, similar to ileus of the small bowel, gastroparesis is a common complication of a wide array of illnesses. The cause of the patient's pulmonary infiltrates, splenomegaly, and liver inflammation remains unclear. While it seems tempting to conclude that her solid organ inflammation was in response to her highly active mycobacterial infection, this has also not been previously described as occurring with leprosy. A concurrent viral infection or viral reactivation syndrome could have caused these findings. Unfortunately, viral titers for cytomegalovirus evaluating for reactivation were not performed. The presence of rhino/enterovirus DNA in respiratory secretions is likely not of clinical significance, as this is a common result amongst patients tested at our hospital regardless of symptoms.

Given the complexity of the case, this report leads us to ponder Occam's razor and then turns to Hickam's dictum: “Patients can have as many diseases as they please.”

## Figures and Tables

**Table 1:** Serum laboratory values at first admission.

Sodium	138 mmol/L	—
Potassium	3.8 mmol/L	—
Chloride	95 mmol/L	Low
Bicarbonate	28 mmol/L	—
Glucose	87 mg/dL	—
Blood urea nitrogen	33 mg/dL	High
Creatinine	1.05 mg/dL	—
Calcium	9.4 mg/dL	—
Protein	12.5 g/dL	High
Albumin	3.70 g/dL	Low
Bilirubin (total)	0.9 mg/dL	—
Alkaline phosphatase	139 units/L	—
Alanine transaminase	162 units/L	—
Aspartate transaminase	138 units/L	High
Lipase	217 units/L	High
White blood cell count	15.1 × 10^3^/mcL	High
Red blood cell	3.89 × 10^6^/mcL	—
Hemoglobin	12.2 g/dL	—
Platelet count	290 × 10^3^/mcL	—
Segmented neutrophils (%)	87.80	High

## Abbreviations

AFB: Acid-fast bacilli
*M. leprae*: *Mycobacterium leprae*
US: United States
CT: Computed tomography
MTB: *Mycobacterium tuberculosis*
PCR: Polymerase chain reaction.
